# The Alternative Factors Leading to Replication Crisis: Prediction and Evaluation

**DOI:** 10.1177/0193841X241229106

**Published:** 2024-02-20

**Authors:** Gregory Chernov

**Affiliations:** 1 Department for Computational Neuroscience, 28328 Max Planck Institute for Biological Cybernetics, Tübingen, Germany

**Keywords:** reproducibility, replications, prediction markets, brier score, C18, C90

## Abstract

Most existing solutions to the current replication crisis in science address only the factors stemming from specific poor research practices. We introduce a novel mechanism that leverages the experts’ predictive abilities to analyze the root causes of replication failures. It is backed by the principle that the most accurate predictor is the most qualified expert. This mechanism can be seamlessly integrated into the existing replication prediction market framework with minimal implementation costs. It relies on an objective rather than subjective process and unstructured expert opinions to effectively identify various influences contributing to the replication crisis.

## Introduction

Experimental research had formed a solid basis in the natural sciences, however, its application in the social and humanitarian sciences has caused significant challenges. The feasibility and reliability of experimental research designs in these fields have been debated and scrutinized (Ioannidis, 2005b; Simmons et al., 2011). In response to the raised concerns, the scientific community has witnessed several significant large-scale replication efforts (Benjamin et al., 2018; Open Science Collaboration, 2015; Ioannidis, 2005a). These endeavors aimed to assess the reproducibility and generalizability of findings derived from prior studies. The effectiveness of these replication efforts themselves is still under discussion and requires evaluation (Laws, 2016; Nosek & Errington, 2017).

An essential portion of replication discussion revolves around the issue of “systematic error” (Schmidt, 2009). This phenomenon occurs when an effect is falsely attributed to a specific aspect of an experiment, whereas it is actually caused by another aspect (Feest, 2019). Feest, in particular, posits that there is a perpetual risk of systematic error due to the potential existence of overlooked confounding variables. Chen and Risen (2010) provide an empirical illustration of systematic error persistence in subsequent replications.

In this methodological essay, we address the claim that replication is overrated (Soler, 2011) due to the intractable systematic error and intrinsic assumptions, that “replication alone is not sufficient to establish internal validity.” We will demonstrate that, indeed, it is necessary yet perhaps insufficient to establish the internal validity of an effective successful replication. Moreover, we will show that expertise is always required in this process, because only established experts can track back the factors leading to replication failure. The problem of tracking those factors is more tricky than it looks because besides poor scientific practices and incentives issues, other factors (like effect heterogeneity) may also play a role. We will provide a taxonomy of these factors and propose a criterion for objective assessment of the current expertise level. This criterion will be founded on assessing the accuracy of predictions made by experts, enabling us to identify and trace the underlying causes of non-reproducibility.

## Systematic Error is Inaccuracy in What?

When some researchers (e.g., Feest (2019)) say that replication is overrated, it is unclear which particular aspects are overrated. This is often left out of discussion, but when mentioned, it is usually implied that a high level of replicated studies does not provide high validity (Machery, 2020). While validity is often used simply as a synonym for reliability and trustworthiness, here we will rely on a more rigorous term definition elaborated (Campbell & Cook, 1979) into four related components. It includes statistical conclusion validity, internal validity, construct validity, and external validity (see all definitions in Table 1).

**Table 1.** Taxonomy From Campbell & Cook (1979).

Type	Description
Statistical conclusion validity	The validity of inferences about the correlation (covariation) between treatment and outcome.
Internal validity	The validity of inferences about whether observed covariation between A (the presumed treatment) and B (the presumed outcome) reflects a causal relationship from A to B as those variables were manipulated or measured.
Construct validity	The validity of inferences about the higher order constructs that represent sampling particulars.
External validity	The validity of inferences about whether the cause–effect relationship holds over variation in persons, settings, treatment variables, and measurement variables.

Now we can restate the basic issue of systematic error in terms of validity: a high replication level would indicate a high level of statistical validity, but not a high level of internal validity.

When the replication rate is low, it becomes difficult to detect any effects, both valid and occasional, as the set of all detected valid effects is encompassed within the set of all detected effects. Therefore, a high percentage of successfully replicated studies becomes crucial for identifying valid effects.

However, it is important to note that the replication of an effect alone cannot be considered a sufficient condition to establish its internal validity, as systematic errors can still influence the results. Likewise, the replication failure cannot reliably refute the presence of an effect. Despite this, substantial number of replicated effects suggests that a significant proportion of studies showcase statistical effects, among which causal effects are likely presented. Hence, achieving a high level of replication across all studies is a necessary condition to enhance the overall internal validity of a research. In essence, without adequate replication, our ability to discover and understand effects is severely limited.

When we switch our attention to a highly coveted but challenging issue of external validity, it becomes clear that the two preceding validities are necessary conditions for its attainment: not for each particular effect, but rather in general for all the discovered effects. Statistical validity, measured by the replication level, ensures the effects detection, while internal validity ensures that these effects are accurately represented as causal relationships. Ultimately, external validity guarantees stability and generalizability of these effects.

Indeed, replication alone cannot address all research challenges. Both replication and distinguishing types of validity developed as a reaction to Fisher randomization in experiments. Since replication should provide evidence of the effect’s stability, it is interesting to track that validity topology arose to eliminate the “erroneous impression that randomization took care of all threats to validity” (Campbell, 1986). Therefore, internal validity by definition cannot be tested by replication. Nevertheless, since replication is still required in the next part, we will look into causes preventing high replication level.

## Non-Reproducibility is Lack in What?

Historically, a lower-than-expected level of replication has been closely associated with the concept of “researcher’s degrees of freedom.” This concept has sparked discussions across various fields, including psychology (Simmons et al., 2011), statistics (Steegen et al., 2016), and economics (Camerer et al., 2016). It draws parallels from mechanics and statistics, applying it to the multitude of choices researchers make at each stage of the research process, including hypothesis formulation, study design, data collection, analysis, and reporting. Different choices made by researchers can result in diverse processed datasets and subsequently yield different statistical outcomes (Wicherts et al., 2016). See Table 2.

**Table 2.** The Researchers Degrees of Freedom Checklist From Wicherts et al. (2016).

Code	Type of Degrees of Freedom
Hypothesizing	
H1	Conducting explorative research without any hypothesis
H2	Studying a vague hypothesis that fails to specify the direction of the effect
Design	
D1	Creating multiple manipulated independent variables and conditions
D2	Measuring additional variables that can later be selected as covariates, independent variables, mediators, or moderators
D3	Measuring the same dependent variable in several alternative ways
D4	Measuring additional constructs that could potentially act as primary outcomes
D5	Measuring additional variables that enable later exclusion of participants from the analyses (e.g., awareness or manipulation checks)
D6	Failing to conduct a well-founded power analysis
D7	Failing to specify the sampling plan and allowing for running (multiple) small studies
Collection	
C1	Failing to randomly assign participants to conditions
C2	Insufficient blinding of participants and/or experimenters
C3	Correcting, coding, or discarding data during data collection in a non-blinded manner
C4	Determining the data collection stopping rule on the basis of desired results or intermediate significance testing
Analyses	
A1	Choosing between different options of dealing with incomplete or missing data on ad hoc grounds
A2	Specifying pre-processing of data (e.g., cleaning, normalization, smoothing, motion correction) in an ad hoc manner
A3	Deciding how to deal with violations of statistical assumptions in an ad hoc manner
A4	Deciding on how to deal with outliers in an ad hoc manner
A5	Selecting the dependent variable out of several alternative measures of the same construct
A6	Trying out different ways to score the chosen primary dependent variable
A7	Selecting another construct as the primary outcome
A8	Selecting independent variables out of a set of manipulated independent variables
A9	Operationalizing manipulated independent variables in different ways (e.g., by discarding or combining levels of factors)
A10	Choosing to include different measured variables as covariates, independent variables, mediators, or moderators
A11	Operationalizing non-manipulated independent variables in different ways
A12	Using alternative inclusion and exclusion criteria got selecting participants in analyses
A13	Choosing between different statistical models
A14	Choosing the estimation method, software package, and computation of SEs
A15	Choosing inference criteria (e.g., Bayes factors, alpha level, sidedness of the test, corrections for multiple testing)
Reporting	
R1	Failing to assure reproducibility (verifying the data collection and data analysis)
R2	Failing to enable replication (re-running of the study)
R3	Failing to mention, misrepresenting, or misidentifying the study pre-registration
R4	Failing to report so-called “failed studies” that were originally deemed relevant to the research question
R5	Misreporting results and *p*-values
R6	Presenting exploratory analyses as confirmatory (HARKing)

Since problems in institutional factors and incentive structures have not been sufficiently explored by the scientific community itself, in our view this is what has led to a focus shift towards controlling replication, for example, through pre-registration. As an example of this perception, we can consider the degrees of freedom checklist from Wicherts et al. (2016). All the items from this list boil down either to biasing institutional practices or to the bad individual disclosure where the researcher misreports methods in a manner that they explain or illusionary lead the results (rather than disclose the real reasons driving that choice). An example of the first point would be publication bias, and an example of the latter would be HARKing. In our view, however, these categories are not the only reason why the level of reproducibility may be unsatisfactorily low in different domains (e.g., in psychology 35 (36%) of the 97 (Open Science Collaboration, 2015), in medicine 20 (44%) out of 49 (Ioannidis, 2005b)). To look more closely at alternative reasons, consider the following scenarios in the Table 3.

**Table 3.** Different Scenarios of Replication Failure.

#	Keyword	Description	Is the Effect Statistically Valid?	Example
1	The fallacy of a single cause	The experiment is designed to measure the effect of incentive A on outcome B. In reality, outcome B is also affected by another variable C, which is not recorded in the initial study protocol and can be randomly changed in subsequent replications.	No, In the presence of effect A → B when A diminishes B and C = 0 in the first experiment. In the second experiment C could be equal to 1 and thus eliminates the AB effect	In Sorge et al. (2014) discovered that the unmeasured variable presence of a male experimenter causes stress and related analgesia in rodents, which potentially lead to the possibility of contamination with past mice related experiments.
2	Heterogeneous treatment effect	The experiment is designed to measure the effect of stimulus A on outcome B. In reality, outcome B depends on the individual’s cohort, and unmeasured variation cohorts in a sample may introduce bias.	No, if there is no registration of cohorts. In the presence of effect A → B only for individuals in the C1 cohort and absence for those in the C2 cohort, the cohorts variation in original and replicated samples would lead to the bias	Watts et al. (2018) were able to refute the findings of the famous marshmallow test. The test argued that a child’s ability to refuse a stimulus for a time (reflecting his self-control) in order to receive a larger reward was related to his later level of success. The authors of the study showed that self-control is related to the socioeconomic status of children (what in fact influences future success), and as a consequence, the results may vary depending on how many of the sample came from affluent families.
3	Randomization failure	Sometimes, from a statistical perspective, it may be necessary to assign a greater number of individuals to the treatment group compared to the placebo group at a ratio of two to one (2:1). However, when pooling data with different allocation ratios, corresponding adjustments need to be made in the statistical analyses. Otherwise, it can introduce bias.	No, the effect can be sufficiently biased and might be not reproduced with a normal 1:1 ratio	In Vorland et al. (2021) an example of a trial examining the effects of weight loss on telomere length in women with breast cancer was provided, where data were combined from two distinct phases of a randomized controlled trial (RCT), each having different allocation ratios.

The aforementioned scenarios differ from the degrees of freedom concept as they do not involve inappropriate incentives or behavior, but rather stem from lack of expertise at the individual or domain level. Scenario 3, for instance, exemplifies a situation where an accepted standard in a given domain is not strictly implemented when needed and therefore represents a low standard example. Standards themselves can also be flawed, such as utilizing Neyman-Pearson Type I error instead of Fisherian error (Rubin, 2021), combining blocking results into a single regression (Pashley & Miratrix, 2022), or the case of a dead salmon appearing alive on tomography due to incorrect measurement aggregations (Bennett et al., 2009). These cases demonstrate that even when researchers adhere to domain-level standards, reproducibility may not be achieved.

Scenarios 1 and 2 share similarities with systematic error as they stem from an incorrect underlying experiment or measurement model. The causal structure differs from what researchers originally envisioned modeling the problem. However, unlike systematic error, problems arising from unmeasured heterogeneity, or the presence of other unmeasured causes can impede reproducibility.

Therefore, lack of reproducibility can be attributed to both the researcher’s degrees of freedom and expertise deficiency at the individual and domain levels. The varying levels of reproducibility observed across different fields within the social and cognitive sciences suggest that lack of expertise can significantly influence the outcome. Without addressing the expertise issue, significant improvements in reproducibility are unlikely to be achieved. Consequently, it becomes challenging to determine from an external standpoint whether the measures taken to counteract degrees of freedom, such as pre-registration, are ineffective in addressing the issue or if the reasons for non-reproducibility extend beyond the scope of degrees of freedom. Further, we provide a possible conceptual solution.

## Prediction of Replication Outcome as Criterion

### Why a Replication Outcome Prediction Could be Useful for Identifying and Tracking a Lack of Expertise

ow can expertise be measured objectively, without relying on others’ expertise (and, therefore, groupthink)? Experts may overstate or understate their expertise and it is hard to provide objective feedback. We can, however, develop such measurement through an objective process—making predictions about unknown future events, verifying whether the prediction is correct, and updating the resulting measure of expertise step-by-step. In this section, we will develop a more specific procedure starting from a highly stylized thought experiment.

Imagine several experts claiming they can color a map using three colors without neighboring countries sharing the same color, but they will not disclose either the entire coloring or their used method to make it. These experts may be incorrect or dishonest and the observer wants to test the experts to learn which of them are genuine. A simple procedure can do that with high confidence. The experts are instructed to place colors assigned to each country in separate sealed envelopes with country names on them. The observer randomly opens the expert’s envelopes for two neighboring countries, checking that the colors differ. This process is reiterated, while experts privately recolor the map with a randomized palette during each iteration (so the observer cannot reconstruct entire coloring from pieces). With each take, the observer’s confidence in identifying true experts grows. But if two envelopes contain the same colors, it suggests the expert is deceptive or erroneous. By conducting a significant number of repetitions, the observer can identify the real experts with a high level of confidence.

The main takeaway from this example is that to make a test we need to collect specific statements which, when verified at random, would enhance an expert’s credibility. To establish such test we can use the replication framework feature. Replication yields a binary outcome as a result for each study; therefore, all properties of random binary outcome processes can be used. When a certain amount of outcomes has been received, the empirical frequencies are used to approximate this random process, which is called a calibrated prediction (Foster and Vohra, 1997). This property can be inverted—an improvement over the calibrated prediction cannot be achieved without the knowledge of the data-generating process (Olszewski, 2015). In our case, it means that the prediction accuracy of a particular expert, which on average passes the threshold of the calibrated forecast, can be explained only by expertise. Consequently, we will use replication outcomes as an objective process (analog of a map with colors), and the prediction accuracy of the expert above the calibrated prediction as an analog of a test (opening envelopes).

Consider an analogy to a map coloring thought experiment applied in the context of social sciences illustrated in Table 4. In this example, we have three types of experiments denoted as A, B, and C. Two of these experiments have not been replicated, while one has, with a natural replication rate of 33%. Let us assume there are two forecasters, one of whom recognizes that an experiment which outcomes theoretically depends on variable X (e.g., consider self-control, which is influenced by students’ sleep patterns. These sleep patterns, in turn, are influenced by the timing of the experiments, particularly when they are conducted in close proximity to examination weeks, which is observed from the dates. This situation introduces an additional, yet unmeasured variable, hence the experts track the dates when doubt the replication.)

**Table 4.** Social Science Analog of Map Coloring Thought Experiment.

#	What was Assumed by Authors	What was in Reality	One of Possible Description	Result of Experiment	Result of Replication	Possibility To Predict By Expertise
A	T → Y	*T* → *Y*←−−*X*	It was assumed that the payment schemeT affects the time preferences Y	Effect presence	No, not replicated, since the experimentswere influenced by X (e.g., sleep levels);decreased sleep during exam weeksin students increased the effect. Real effectworks only when X is also active	Yes, (in the example the experiments dates were known)
B	T → Y	T → Y	It was assumed that the observation of averagechoices T affects Y—the other regarding preferences	Effect presence	Yes, replicated	No
C	T → Y	*T Y*←−−*X*	It was assumed that the power posingT affects the testosterone level Y	Effect presence	No, not replicated	No

Further, let’s assume that each of these experiment types has been conducted 1000 times, and both forecasters are perfectly calibrated (the implications of this assumption will be elaborated upon in the following section). In this scenario, if the expert forecaster, who is aware of X influencing Y in experiments of type A, consistently predicts their outcomes, their average prediction accuracy will be 66%. On the other hand, the forecaster who predicts only half of them will achieve an average accuracy of 50%, which identifies the true expert.

The approach of predicting replication outcomes has been previously explored as a means to reduce replication costs (Camerer et al., 2016; Dreber et al., 2015) and assess the quality of expertise (DellaVigna & Pope, 2018). To serve as a viable test criterion, it must effectively identify genuine experts and align with the parameters employed in replication studies.

To address these replication parameters, we will employ a framework (Maniadis et al., 2014). Subsection “Forecasting Task in a Replication Framework” will delve into the detailed workings of this framework.

To ensure that the expert predictor is not merely a charlatan using calibration rules to generate seemingly accurate forecasts without possessing genuine underlying information we use Foster & Vohra (1997) criterion. In subsection “Using Calibration as a Criterion to Reveal True Expert”, we will outline how calibration can serve as a distinguishing criterion, similar to the envelope color match. By using calibration as a metric, we can evaluate the expert’s performance in detail.

Our final goal is to establish more robust scientific institutions: subsection “Default Minimal Simple Procedure” will propose a comprehensive procedure for identifying potential causes of non-reproducibility. This procedure actively leverages the principle that the most accurate predictor is the best expert for analyzing the cause. Thus, experts who successfully predict outcomes and are authorized to be true experts can also weigh in on the factors they believe to be the primary causes of non-reproducibility. By aggregating the input of genuine experts, we can compile an authoritative list of reasons behind the replication failure.

### Forecasting Task in a Replication Framework

A unit of a forecasting task is an expert’s disclosure statement, which in the replication framework is a forecast for each binary outcome (will the study be reproduced or not). To ensure accurate calculations in subsequent steps it is important to include all parameters impacting the replication outcome into a forecast.

Given the sample size, type I (called *α*), and II (called *β*) errors are fixed, the probability experiments’ replication *R* of *N* experiments is equal to the number of true discovered associations divided by all the associations declared “true”: *RR* = *TP*\(*TP* + *FP*), where RR—replication rate, TP is a true positive (1 − *β*), and FP is a false positive (*α*). Thus we could separate two types of forecast for a single experiment: *π*_*i*_ is given by expert subjective probability that experiment *i* will replicate, $\hat{y_{i}}=p\left(\pi_{i}|RR\right)$ is a subjective probability including a prior. According to Maniadis et al. (2014), it could be calculated as:

(1) $$\hat{y_{i}}=\frac{\left(1-\beta_{i}\right)\pi_{i}}{\left(1-\beta_{i}\right)\pi_{i}+\alpha_{i}\left(1-\pi_{i}\right)}$$

Whether a prior is taken into account when we ask an expert to make a forecast remains a question. The difference is whether we need to adjust the reported probability from *π* to $\hat{y_{i}}$ . On the one hand, the experts are familiar with experimental protocol and their forecast already takes into account all the information. On the other hand, human reasoning is far from an accurate Bayesian updating especially when different experiments included in the replication have different *α* and *β*.

### Using Calibration as a Criterion to Reveal True Expert

With a precise prediction in hand, we can now utilize the results of the replication to determine whether the effect is reproducible. This involves examining whether the effect magnitude falls within the confidence interval established in the initial study. By doing so, we can establish a criterion for distinguishing between genuine experts and charlatans.

The experts quality in forecasting tasks has to be evaluated by predictive metrics, so the choice of metric matters. The task organizer could use any arbitrary measure as a rule which satisfies the requirement of proper scoring rules (Gneiting & Raftery, 2007). Any proper scoring rule counts the weighted aggregate of mistakes, they differ in what aggregate is used (sum, product, or something else) and in the weighting function for those mistakes. Building upon the work of Dreber et al. (2015), we adopt the Brier score as the foundation for our analysis. The Brier score is commonly used for evaluating binary outcomes, where the response variable, denoted as *y*, takes values of either 0 or 1:

(2) $$\text{BS}=\frac{1}{N}\overset{n}{\underset{i=1}{\sum }}\left({\left(\pi_{i}-y_{i}\right)}^{2}+{\left(y_{i}-\pi_{i}\right)}^{2}\right)$$

Here *π* is taken as a base, but it might be replaced with $\hat{y}$ if experts still have not taken priors into account. Thus as soon as all forecasts are reported and all experiments are replicated the most accurate forecasters will be identified by one of the scoring rules. When we are only interested in the experts accuracy, it will be enough to incentivize them and rank by Brier. However, we want not just to estimate their accuracy, we aim to distinguish an actual “expert” from an ignorant but well-calibrated forecaster.

To illustrate this idea, consider an example from Foster and Hart (2021). Let outcomes alternate in a deterministic way from success to failure y = {0, 1, 0, 1… 0, 1}. If we compare 3 types of predictions *p*_1_ = {0, 1… 0, 1}; *p*_2_ = {0.1, 0.9, … 0.1, 0.9}; and *p*_3_ = {0.5, 0.5, … 0.5, 0.5} we will see that while the first forecast is the ideal the rest have different properties. The third is ideally calibrated however it is not capable to distinguish between positive and negative outcomes. The second, however, is almost ideal but it isn’t calibrated. Now, from the prior replication ratio RR and empirical frequency of replication— $\bar{y}$ of an arbitrary replication study, we can define an ex-post difference $\delta =RR-\bar{y}$ . We are interested in separating good forecasters who are capable to accurately identify positive and negative outcomes but are mistaken about *δ* size from those who just guess the *δ* successfully but have no expertise in non-replicated studies. We propose two possible ways to resolve this issue. The first is to use Brier decomposition. The second is to adjust Brier by a benchmark artificial forecaster with manually determined parameters.

Let’s consider them one by one, with Brier decomposition determined as

(3) $$BS_{dec}=\frac{1}{N}\overset{K}{\underset{k=1}{\sum }}n_{k}{\left(\pi_{k}-{\bar{y}}_{k}\right)}^{2}+\frac{1}{N}\overset{K}{\underset{k=1}{\sum }}n_{k}{\left(y_{k}-{\bar{y}}_{k}\right)}^{2}$$

where $\bar{y}=\overset{N}{\underset{t=1}{\sum }}y_{t}/N$ is the total empirical frequency of replicated outcomes among all outcomes, *n*_*k*_ indicates the number of observations in each response category, ${\bar{y}}_{k}$ percentage of correct answers for each probability category given forecasts of probability *π*_*k*_. The first component in the sum is called calibration and the second is called refinement, thus *BS*_*dec*_ = *CAL* + *REF*. For our examples forecast *p*_1_ has *CAL* = 0 and *REF* = 0, forecast *p*_2_ has *CAL* = 0.01 and *REF* = 0.01, forecast *p*_3_ has *CAL* = 0 and *REF* = 0.25. Hence, to incentivize the forecaster and to recognize the true expert, they need to be stimulated by their refinement score.

The second approach starts with a calculation of Brier skill score (BSS), which takes as a benchmark *BS*_*ref*_—some other forecast: $BSS=1-BS/BS_{ref}$ . Naturally, we will take as this benchmark the ideal ex-post calibrated score:

(4) $$BSS=1-\frac{BS}{\frac{1}{N}\sum_{i=1}^{N}\left({\left(\bar{y}-y_{i}\right)}^{2}+{\left(y_{i}-\bar{y}\right)}^{2}\right)}$$

The Brier score serves as a loss function, where a lower score indicates better performance, with a perfect score of 0 being the optimal outcome. However, when considering the Brier skill score, a higher value is desirable, with 1 (or 100%) representing the best possible score. In the context of separating an expert from a calibrated model, our criterion is based on the expert’s Brier skill score going below zero. A negative Brier skill score indicates that the expert’s performance is worse than the calibrated model. Therefore, the experts can only outperform the calibrated model if they possess expertise or knowledge about the underlying process, which aligns with our objective in this study.

### Default Minimal Simple Procedure

To identify the factors contributing to replication failures, we propose a straightforward procedure. First, we gather a pool of candidate studies for replication and invite researchers to participate as volunteer forecasters.

Next, the forecasters are given surveys where they allocate 100 scores across the factors. Factors include both factors from individual and domain cohorts and all factors from the Table 2. Experts are asked to distribute the scores based on the perceived importance of each factor in predicting non-replication for each study in the replication pool. Additionally, they provide replication likelihood forecasts for each hypothesis, rating the probability of replication on a scale from 0% to 100%. Participants are incentivized based on their performance using Brier skill scores, which reward accuracy in forecasting. After getting the results we can calculate a score of each factor and rank it according to its association with replication failures:

(5) $$FS_{j}=\overset{M}{\underset{m=1}{\sum }}\left(\overset{N}{\underset{i=1}{\sum }}\left[\frac{w_{ij}}{\sum_{k=1}^{J}w_{ik}}\times BSS_{m}\times \left(1-{\hat{y}}_{im}\right)\right]\right)$$

where $w_{j}/\sum_{0}^{J}w_{i}$ is a reported weight (factor score divided by 100 in total) of a factor among other reported factors, m, i, j denote indexes of tested experts, studies, and factors, respectively, and ${\hat{y}}_{im}$ is a forecast.

Through this procedure we aim to pinpoint and prioritize the factors that play a role in non-reproducibility within scientific studies. By assessing the Brier skill scores (BSS) of each individual, we can distinguish the true experts from the rest. This allows us to focus solely on the scores provided by those who have BSS higher than 0 and thus demonstrate the expertise in the field.

## Discussion and Applications

The issue of non-reproducibility in scientific studies is a complex one, due to systematic errors and confounding variables (Crandall & Sherman, 2016; Feest, 2019). Mere success in replicating an individual study does not guarantee indisputable conclusions, challenging the benefits of replication. Recent discussions (Hudson, 2023) have focused on the concept of indirect replication as a potential solution, aiming to address internal validity concerns.

In this essay, it has been argued that replication and validity are not directly linked as various factors beyond causality can contribute to irreproducibility. A conceptual solution for tracking these factors has been proposed, along with an implementation framework. The framework is relatively simple as it utilizes existing replication prediction markets (Camerer et al., 2016; Dreber et al., 2015) with an addition of the questionnaire for participating experts.

To implement the proposed approach, it is advisable to integrate it into existing initiatives rather than create separate replication projects. If the integration of calibration exercises into standard practices within the social sciences becomes more widespread, it could usher in a significant transformation of research conduction and evaluation.

Large-scale replication projects have already been accompanied by ones that predict their outcomes. For instance, in the field of experimental economics, we have initiatives like the Experimental Economics Replication Project (EERP, Camerer et al. (2016)), the Social Science Replication Project (SSRP, Dreber et al. (2015)), and smaller-scale projects conducted by individual labs, such as WKW, DellaVigna and Pope (2018). These projects require collaboration among several labs yet not an overwhelmingly high number of participants: EERP (involving 18 authors, 18 studies, and 97 experts), SSRP (involving 8 authors, 44 studies, and 52 experts), and WKW (involving 2 authors, 15 studies, and 208 experts). The ultimate goal of these additional calibration exercises is multifaceted. Firstly, they aim to untangle the complex web of factors contributing to non-replicability, distinguishing between social and domain-specific influences. Secondly, these exercises demonstrate the potential for enhancing replication forecasts through the expertise of seasoned researchers. Lastly, they aim to provide open-source materials in the form of checklists to assist researchers in meticulously planning and executing their studies.

This is only a transitional solution, yet a necessary step that acknowledges the complexity of the issue. A deeper understanding of reproducibility reasons and establishing expert ratings may result in a protocol for full-fledged conceptual replication.
